# Biographical articles in scientific literature: analysis of articles indexed in Web of Science

**DOI:** 10.1007/s11192-018-2923-3

**Published:** 2018-10-05

**Authors:** Olesia Iefremova, Kamil Wais, Marcin Kozak

**Affiliations:** Department of Quantitative and Qualitative Methods, University of Information Technology and Management in Rzeszow, Sucharskiego 2, 35-225 Rzeszow, Poland

**Keywords:** Authors, Biographical articles, Citations, Gender, GenderizeR, Document type

## Abstract

Biographical articles in scientific journals offer a platform for the commemoration of distinguished individuals from the world of science. Despite so important a role for the scientific community, research on biographical articles is scarce. To fill this gap, we have analyzed 190,350 biographical articles indexed in Web of Science, written by 251,908 authors in 1945–2014. We have analyzed the development of this article type over the studied period and research areas, how women and men are represented in the subject of articles, and who the authors are. Over the time the number of biographical articles has been increasing, with the highest number in Life Sciences and Biomedicine. Around 20% of the articles were written about women, with the highest share of 24% in Arts and Humanities. Both male and female authors write more often about men than about women, a stable situation for the last 70 years.

## Introduction

Article types vary in their roles in the dissemination of knowledge and in their frequency in journals (Sigogneau 2000). Articles, normally considered a source of original research, are the most basic—and the most important—document type; for instance, they accounted for 57.4% of all documents indexed by WoS for 2014. Less frequent in WoS, but still important, are proceeding papers (13.4%), meeting abstracts (11%), editorial materials (4.4%), book chapters (4.3%), reviews (3.0%), book reviews (2.6%), and letters (1.7%). All others document types accounted for 2.2% of all the documents in 2014.

Among these infrequent document types are “biographical items” and “items about an individual.” According to WoS, biographical items are “obituaries, articles focusing on the life of an individual, and articles that are tributes to or commemorations of an individual” (Web of Science 2014) while items about an individual are “review of the work(s) of a celebrated person in a particular field of study” (Web of Science 2014). In 1998, the two article types were joined into category biographical item, so we will not differentiate them throughout the whole studied period; we will refer to them by one term, *biographical articles*.

Biographical articles constitute a platform for celebrating lives of distinguished personalities. Such papers pay tributes to honored scholars, artists, and other people who contributed to the development of science or culture. By appreciating those who deserve appreciation, biographical articles, thus, play an important role in merging scientists into the scientific community and in opening this community to the society.

Biographic articles are one of many types of scientific articles. Various types of scientific articles have been studied in scientometrics literature, mainly in relation to their impact on scholarly communication. Examples of such studies are those on review articles (Lewison 2009; Ketcham and Crawford 2007), proceedings papers (Ingwersen et al. 2014; Sigogneau 2000), correspondences (Kozak and Hartley 2013), editorial material (van Leeuwen et al. 2013), and book reviews (Zuccala and van Leeuwen 2011). Biographical articles, however, have not gained much interest in the scientometrics community, a likely reason being their rarity: In 2014, for instance, WoS indexed 4287 biographical articles, accounting for about 0.2% of all documents indexed that year.

This does not mean, however, that researchers have ignored such topics. Ball and Jonnes (2000), Fowler and Bielsa (2007), Starck (2008) and Epstein and Epstein (2012), for example, studied obituaries published in popular newspapers and magazines. While these studies dealt with obituaries in general, some other analyzed those related to academia. Hamann (2016) studied 216 obituaries from academic journals in physics, history, and sociology, published in the USA, the UK, and Germany in 1960–1970, 1980–1990, and 2000–2010. The author collected data on the persons’ scientific discipline, fields of expertise, relation to recognizable researchers in the field, and relationships between the author of an obituary and the deceased. He also analyzed the persons’ PhD and the last university appointment before the death. Hamann—who treats obituaries as the unofficial academic evaluations of the dead by his or her peers—searched the obituaries for the mentions of predetermination of academic success in early life, character traits, hard work, and dedication to academic life.

Tight (2008) analyzed 134 obituaries of 100 academics (15 women and 85 men) published in the UK newspapers (*The Guardian*, *The Times*, *The Daily Telegraph*, and *The Independent*) in 2007 to study contemporary academic work. The mean age was 79 (69 for the women and 80 for the men). Half of the academics were from arts, humanities, and social sciences; the other half, from the sciences and medicine. Sixty academics were born in the UK or Ireland, 15 in other parts of Europe, 14 in North America, and 3 in Asia, Africa, and Australia each. At least 81 were professors. The majority studied or worked in the Oxford or Cambridge university. The sample included five Nobel Prize winners. Several features were described in all the obituaries: family background, education, career, professional and personal qualities, and achievements.

Macfarlane and Chan (2014) studied scholars’ obituaries published in popular media. To analyze the concept of intellectual leadership, the authors analyzed 63 obituaries published from 2008 to 2010 in *Times Higher Education*. They defined an intellectual leader as a person who is “looked upon by others in their discipline or profession, respected and inspiring figure.” The authors used NVivo software for textual analysis of obituaries to find scholarly and personal characteristics that were most common in the obituaries. Based on these characteristics, the authors created a tree map to visualize what it means to be an intellectual leader.

These studies focused on obituaries, but biographical articles constitute a wider category. Here, we analyze it as a whole. In order to do so, we analyze a collection of documents classified by WoS as either biographical item or item about an individual, published from 1945 to 2014 in scholarly journals indexed in Web of Science. Since this is the first analysis of a large collection of biographical articles, we have only indirect hints about what we might look for in these data, hints that result from scientometrics research on other document types as well as on obituaries, whether related to academia or not. We will thus conduct our analyses around the following questions: How did the number of biographical articles change over the years? Do journals representing different scientific disciplines differ in the number of biographical articles they publish? Are women and men equally represented in biographical articles? Do women (men) write more articles about women or men? We will not, however, limit the analysis to these questions, but we will explore any phenomena in the data that catch our eyes. In addition, we will analyze the variety of biographical articles in terms of their contents. WoS defines biographical articles rather generally, so we will investigate what indeed can be found in articles that are classified as biographical items or items about an individual.

## Methods

### Gender analysis

#### Data sources

We searched the Web of Science (WoS) database (Web of Science 2016) for two types of biographical articles, namely, Biographical-Item and Item-About-an-Individual, published from 1945 to 2014. This way, we collected the following data about 190,350 unique biographical articles:

- WoS accession number (the unique identifier of an item),
- title,
- year of publication,
- language of article,
- authors’ names (surnames with first names or initials),
- one or more WoS category of the item, and
- the number of citations.

The first WoS category defined for an item was used to assign a higher-level WoS Research Area to this item.

To classify the authors and people mentioned in titles of articles based on their gender, we used genderizeR package (Wais 2016a) of R (R Core Team 2017). The package guesses the gender of a person based on the first name and the data gathered in the *genderize.io* database (Strømgren 2016). Created in August 2013, the database has been regularly updated since, by the continuous scanning of public profiles of social network users. In April 2014, the *genderize.io* database contained information of about 120,000 first names based on about half a million social network profiles of men and women. Almost 3 years later, in June 2017, the database had information of over 200,000 first names from social network profiles from 79 countries and in 89 languages (Strømgren 2016).

#### Glossary

*Authorship*—the unique combination of the title of an article and the name of one of the authors (note that the same author can publish more than one article, so the number of authorships will be greater than the number of authors).

*Biographical article*—an article assigned to one of the two categories in WoS database: Biographical-Item and Item-About-an-Individual.

*Unisex first name*—a first name that can be used both by men and women.

*Gender database*—a database used for gender classification; in our study, we used *genderize.io* database, which contains information about relationships between first names and gender obtained from public profiles from social networks.

*Probability*—given a first name, a probability that the person with this first name is men (or women, depending on the context). If the probability is 0.5, half of the people in the gender database who share this first name are men while the other half are women.

*Count*—a number of people in the gender database with the same first name.

#### Gender classification

We used the methodology suggested in Wais (2016b) to guess the gender of (i) people mentioned in titles of biographical articles and (ii) authors of these articles. The algorithm, available in the genderizeR package (Wais 2016b),

1. automatically parses all title words,
2. checks in the *genderize.io* database if these words were used as first names in social network profiles, and
3. estimates probability that a person with this first name is men or women.

In the third step above, the algorithm takes into account that some first names are valid for both men and women, and so classifying such names is always imprecise. Using the gender data from the database, we can estimate this uncertainty: given a first name, the probability of being a woman is estimated as the share of people with this first name who declared themselves as women.

#### Validation of gender classifications

*Validation datasets* We validated the algorithm with a random sample of 1000 unique biographical articles. The gender of persons in the titles were manually coded as

- “male” or “female”, if all people mentioned in the title had the same gender,
- “both”, if more than one person was mentioned in the title and their gender was different,
- “unknown”, if it was impossible to assign a gender based on the name given in the title, or
- “noname”, if no person was mentioned in a title.

This way, we coded the gender of persons in the titles as
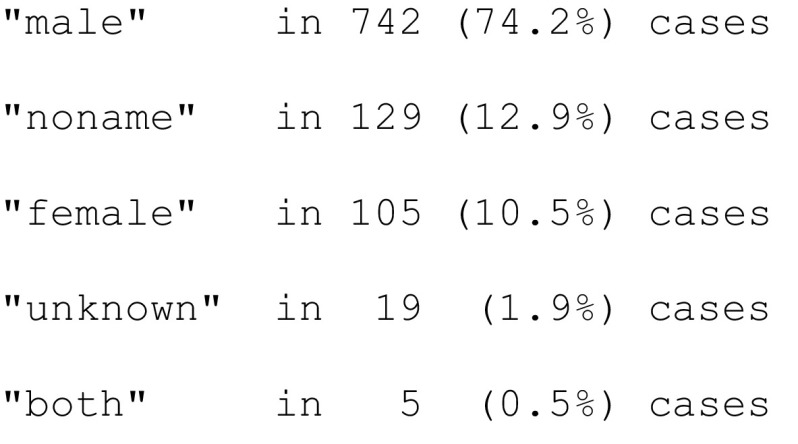


Similarly, to validate how precisely the algorithm classified the gender of authors, we randomly sampled 2000 biographical articles and extracted 2641 author names. If the first name of an author was given, the author’s gender was manually coded as a “female” or “male,” based on Internet queries that used the author’s affiliation, contact information, and the title of the biographical article. We coded the gender of authorships as
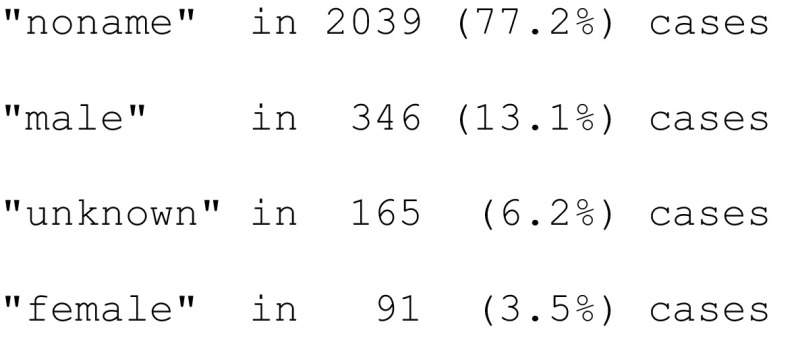


*Training the algorithm* From the *genderize.io* database, for each first name we have probability that a person with this name is man or woman. We have to decide whether we wish to work only with names for which this probability is close to 1 or we accept also names for which this probability is closer to 0.5; for a probability close to 0.5, such a name is given to both men and women, and so classification of the gender for such a name will be the most uncertain.

Thus, to train the algorithm for classifying gender, we should check different threshold values of this probability and choose the best one. The algorithm will not use first names with probabilities below this threshold; this way, we can decrease the uncertainty of our classifications at the cost of ignoring unisex first names.

We should also be cautious when using rare unisex first names. To decide which names should be included in the algorithm and which ignored, we should test different threshold values for counts of how many times a first name was recorded in the gender database; the algorithm will use only those first names which occurred more often than the threshold.

So, we looked for the optimum values of these two parameters: probability (that a first name represents a particular gender) and count (of how many times a first name was recorded in the database with gender data) (Wais 2016b). Based on a preliminary, exploratory analysis, we have decided that the optimum probability should be between 0.5 and 0.8 while the optimum count, between 1 and 13. Note that the algorithm should be independently trained for the two datasets: titles and authorships. For both datasets, we checked all 403 combinations of (i) probability between 0.50 and 0.80 with a step 0.01 (so, 0.50, 0.51, … , 0.80) and (ii) count between 1 and 13. The best combination is that which leads to the highest accuracy of gender classification, that is, for which the algorithm would match the manually coded data in the highest number of cases.

For the validation dataset of titles, the algorithm worked best with the probability parameter set to 0.67 and the count parameter set to 1. Using these values, we obtained a relatively small overall classification error rate (8.7% percent of items with incorrectly classified gender) and a small proportion of items with an unclassified gender (1.9%). The gender bias error rate in automatic gender classification was also low (4.1%) and had a positive sign, which suggests that more men were incorrectly classified as women than vice versa, indicating a slight overestimation of the proportion of women in the population studied. Since we estimated the overall classification error rate (8.3%) on the training dataset, the error was underestimated. Thus to get a more realistic indicator of classification error rate, we also estimated a more robust bootstrapped error rate (8.5%) (Wais 2016b).

For the validation dataset of authorships, the algorithm worked best with the probability parameter set to 0.54 and the count parameter set to 1. Using these values, we obtained small overall classification error rate (6.9% and bootstrapped error rate 7.1%), small proportion of items with unclassified gender (2.7%), and small gender bias error (1.4%).

### Categories of biographical articles

#### Terminology

Web of Science defines *biographical items* and *items about an individual* (which we join to a document type of *biographical articles*) as, generally put, articles focused on life of individuals, obituaries, tributes, and commemorations as well as tributes to such people. The latter group represents articles that are *not* considered biographical in the traditional meaning; these can be, for example, transcripts of lectures or review articles on a given topic, whose only relation to an individual is dedication of the article.

Individual biographical articles, thus, can differ quite a lot. Thus, we conducted an in-depth analysis of a sample of 750 biographical articles, to find out whether they can be classified into distinct categories. After a preliminary analysis, we divided the articles into those about alive and dead people. We divided these categories into subcategories based on the purpose of an article (Table 1).

**Table 1:** Categorization of biographical articles

Subcategory	Explanation/examples
*Category: Article in honor of an individual (alive)*	
Celebration of work	A review of achievements of an individual; an article about a new member of an association; the appointment of an individual to an important position
Anniversary of birthday	An article about an individual on his or her birthday and/or a review of his or her achievements
Award for individual	An article about an individual who received a scientific or industry award for his or her work
Autobiographical article	An autobiographical article written by a scientist
*Category: Article in honor of an individual (deceased)*	
Obituary	An article about the recent death of an individual
Celebration of work	A review of achievements of an individual and his or her impact on the field. Usually, such articles are about historical figures
Anniversary of birthday	An article about an individual on an anniversary of his or her birthday. Usually, such articles are about historical figures
Anniversary of death	An article about an individual on an anniversary of his or her death. Usually, such articles are about historical figures
*Category: Other*	
	An article that does not fit any of the above subcategories
*Misclassified articles*	
	This is not a category of biographical articles. Misclassified articles are those which have nothing to do with biographical articles and yet were incorrectly classified by WoS as such

We decided to create a special category for atypical biographical articles, which we called “Other.” It would include, for instance, articles that are not about any individual but are dedicated to a person. An example is tributes explained above. Another example could be an article that is focused on a scientific knowledge, with additional explanation of people who developed this knowledge—although such an article includes the biographies of these people, this topic is additional to the main topic. We decided such articles of marginal biographical character should fall into a different category than those which are biographical in their essence.

#### Data collection and sources

**Fig. 1:**
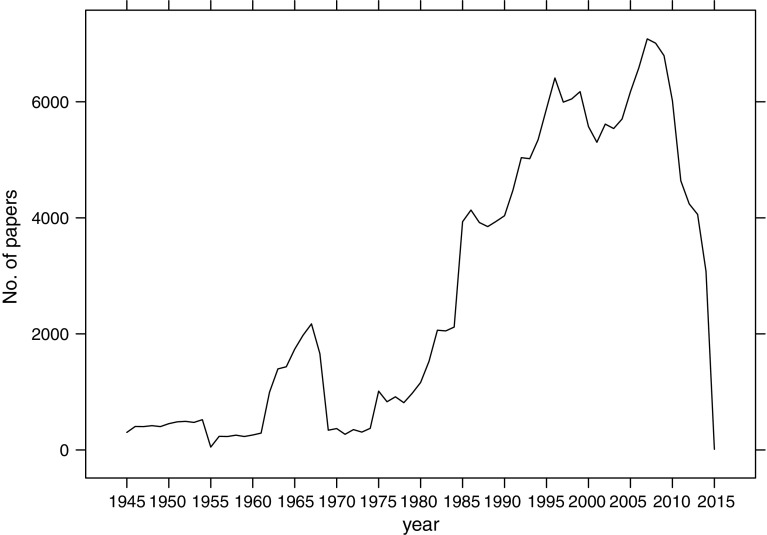
Number of biographical articles published in 1945–2014. *Data source*: Web of Science

We analyzed a sample of 750 biographical articles. To do so, we took three independent subsamples of 250 articles from years 1945–1984, 1985–1999, and 2000–2014, from which we took random samples without replacement. We chose these three periods based on the trend of the number of biographical articles (Fig. 1). The years 1985 and 2000 showed changes in the trend, so we decided to break the whole period into the three corresponding sub-periods and check whether the categorization of biographical articles differed between the periods.

We analyzed each article from the sample in a following manner. First, we looked for particular words in their titles. For instance, articles whose titles included words “obituary” or “in memoriam” were classified as obituaries while articles whose titles contained information about awards given to a person were classified as “award for individual.” Then, we searched for all other articles in WoS, Google Scholar, and/or archives of the journal they were published in. When we succeeded to find their full texts, we read them and assigned a category and subcategory. In some cases, we failed to access full texts but succeeded to classify the articles based on their abstracts or first pages. Sometimes, to reinforce our guesses about classification of an article about this person, we additionally used the information about an individual we found in news articles and press releases.

## Results

As of January 2015 Web of Science indexed information about 190,350 unique biographical articles written by 251,908 authors in the period studied. Below, we analyze those articles and their authors.

### Articles

#### Trends

Of the 190,350 biographical articles from 1945 to 2014, 51.6% were classified as biographical item while 48.4%, as item about individual. Most of the biographical articles were published in *BMJ British Medical Journal* (formerly *British Medical Journal*) (10,572 articles), *Opera News* (2664), *Chemical Engineering News* (2108), *Dance Magazine* (1905), and *Opera* (1782). Across the studied period, the trend in the number of biographical articles published yearly was growing, with a surge in their number between 1962 and 1968. The increase in the number of articles in 1962 can be explained by the appearance of biographical articles in medical journals, like *BMJ British Medical Journal*, *Canadian Medical Association Journal*, *Lancet*, which were scare in preceding years. In 1969 only 340 biographical articles were published. This was caused by a plunge in the number of biographical articles from medical fields. The question remains why there was such a surge in the number of biographical articles published in medical journals and why it ended. Currently, the number of biographical articles is in the phase of decrease, which started in 2007 (Fig. 1). The downward trend can be attributed to the development of the Internet and the transition of the publishing strategy biographical articles from traditional publishing in scientific journals to online publishing on webpages of organizations.

Most biographical articles have been published in Life Sciences and Biomedicine, followed by Arts & Humanities, and Physical Sciences; the fewest, in Multidisciplinary Sciences, Technology, and Social Sciences (Table 2). This corresponds to the higher number of journals and articles published in Life Sciences and Biomedicine. In Art & Humanities, on the other hand, there is a prevalent tradition of introducing new talents in music, ballet, and theater, consequently increasing the number of biographical articles.

**Table 2:** The number of biographical articles in WoS research areas in 2014

WoS research area	No. of articles	%
Life Sciences & Biomedicine	78,406	41
Arts & Humanities	50,048	26
Physical Sciences	24,808	13
Social Sciences	18,290	10
Technology	15,158	8
Multidisciplinary Sciences	3499	2
Total	190,209	100

The articles have been written in 41 languages, with English accounting for 80% of the articles, German for 7%, French for 4%, Russian for 3%, and other languages for less than 2%.

#### Citations

**Fig. 2:**
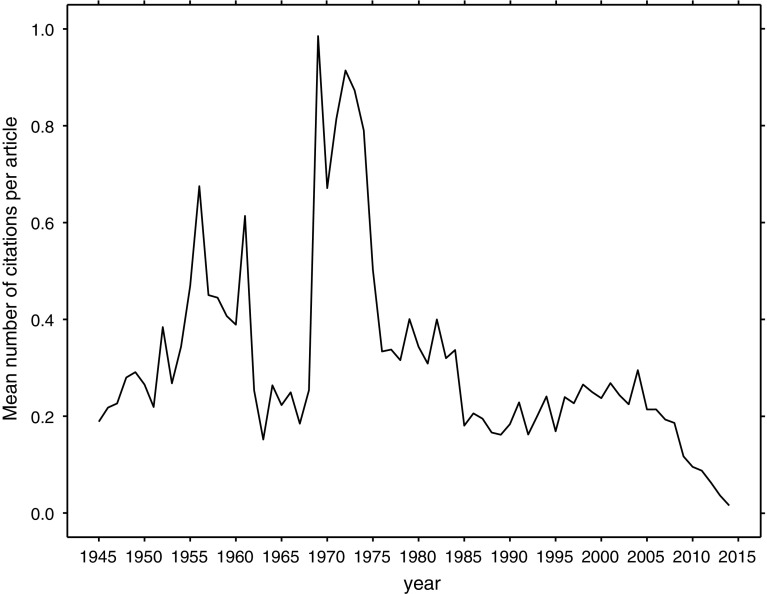
Mean number of citations per biographical article, for articles published in 1945–2014

Although, when calculating impact factor, WoS does not consider biographical articles as citable items, citations to them contribute to the overall number of citations for a journal (Garfield 2006; McVeigh and Mann 2009). Thus, citations of these articles are worth studying. Most of the biographical articles have been infrequently cited or not cited at all; a few, however, have had many citations. The mean number of citations per article for all studied years was below 1 (Fig. 2).

Scientific articles from Social Sciences are usually less often cited than those from Life Sciences and Physical Sciences, a phenomenon we have not observed for the bibliographical articles: those from Multidisciplinary and Social Sciences were most often cited (with the mean number of citations of 0.36 and 0.32, respectively), followed by Life Sciences & Biomedicine (0.27), Physical Sciences (0.25), Technology (0.14), and Arts & Humanities (0.08). The sharp decrease in the mean number of citations per article between 1962 and 1968 may be attributed to the surge in the number of these articles with 290 in 1961 and 995 in 1962. In 1969 there was a five-fold decrease in the number of biographical articles, which corresponded to an increase in their mean number of citations.

We have analyzed top ten biographical articles with the highest number of citations in the Web of Science database (Table 3). All of them were in English, and all but one were assigned to Life Sciences & Biomedicine, Technology, and Physical Sciences research areas. The most cited bibliographical article was Murdoch (1994) with 385 citations; this article was previously presented at a conference in 1991 by population ecologist William W. Murdoch. His lecture was awarded Robert H. MacArthur Award, one of the most prestigious prizes given by the Ecological Society of America (ESA Historical Records Committee 2014). Like Westphal (1975), this article is not a typical biographical article as it is not about an individual—we thus classified it as “Other.”

**Table 3:** Top 10 articles with highest number of citations

Reference to article	No. of citations	WoS category	WoS research area	Type of article from the publisher	Category; subcategory
Murdoch, W. W. (1994), Population regulation in theory and practice. The Robert-H-MacArthur-award-lecture presented August 1991 in San-Antonio, Texas, USA. *Ecology*, *75*, 271–287. 10.2307/1939533	385	Ecology	Life Sciences & Biomedicine	No specific identification about type of article Article is part of MacArthur Award Series An award-winning lecture (Robert H. MacArthur award, 1990) presented at a conference by population ecologist William W. Murdoch	Other
Westphal, O. (1975). Bacterial endotoxins—The Second Carl Prausnitz Memorial lecture. *International Archives of Allergy and Immunology, 49*, 1–21. 10.1159/000231374	194	Allergy; Immunology	Life Sciences & Biomedicine	No specific identification about type of article A lecture by Otto Westphal describing pioneers of bacterial endotoxins, presented at a conference	Other
Eknoyan, G. (2008), Adolphe Quetelet (1796–1874)—The average man and indices of obesity. *Nephrol Dial Transplant*, *23*(1), 47–51. 10.1093/ndt/gfm517	142	Transplantation; Urology; Nephrology	Life Sciences & Biomedicine	Historical note An article about astronomer, mathematician and sociologist Adolphe Quetelet and his research	Article in honor of individual (deceased); Celebration of work
Koppenol, W. H. (1993). The centennial of the Fenton reaction. *Free Radical Biology and Medicine*, *15*(6), 645–651	135	Biochemistry; Molecular Biology; Endocrinology; Metabolism	Life Sciences & Biomedicine	Review article. An article about chemist Henry J. H. Fenton and his research, with the focus on the Fenton reaction	Article in honor of individual (deceased); Celebration of work
Shaw, S., & Pierre, C. (1991). Non-linear normal modes and invariant manifolds. *Journal of Sound and Vibration, 150* (1), 170–173. 10.1016/0022-460x(91)90412-d	126	Acoustics; Engineering, Mechanical; Mechanics	Technology	Letter to the editor. The article does not present any particular individual or event	Misclassified article
Garfield, E. (1982). J.D. Bernal—The Sage of Cambridge. *4S Award Memorializes His Contributions to the Social Studies of Science, Current Comments, 41*, 5–14	125	Multidisciplinary Sciences; Social Sciences, Interdisciplinary	Social Sciences	Essays of an Information Scientist. An article about John D. Bernal, FRS, a pioneer in X-ray crystallography in molecular biology, and his research	Article in honor of individual (deceased); Celebration of work
Noble, D. (2008). Claude Bernard, the first systems biologist, and the future of physiology. *Experimental Physiology, 93*(1), 16–26. 10.1113/expphysiol.2007.038695	100	Physiology	Life Sciences & Biomedicine	Paton Lecture. This article is based on the Paton Lecture delivered with the same title to the Life Sciences 2007 meeting in Glasgow in July 2007	Article in honor of individual (deceased); Celebration of work
Slack, J. M. W. (2002). Timeline—Conrad Hal Waddington: the last renaissance biologist? *Nature Reviews Genetics*, *3*, 889–895. 10.1038/nrg933	96	Genetics; Heredity	Life Sciences & Biomedicine	Perspectives. An article about developmental biologist, paleontologist, geneticist, and philosopher Conrad Hal and his research	Article in honor of individual (deceased); Celebration of work
Tauber, A. I. (2003). Metchnikoff and the phagocytosis theory. *Nature Reviews Molecular Cell Biology*, *4*(11), 897–901. 10.1038/nrm1244	94	Cell Biology	Life Sciences & Biomedicine	Perspectives An article about zoologist and Nobel Prize Laureate Ilya Ilyich Metchnikoff and his research	Article in honor of individual (deceased); Celebration of work
Lutwak, E. (1991). Extended affine surface area. Dedicated to Professor Heinrich Guggenheimer on the Occasion of His 65th Birthday. *Advances in Mathematics, 85*(1), 39–68. 10.1016/0001-8708(91)90049-d	86	Mathematics	Physical Sciences	Original research article. Dedicated to Professor Heinrich Guggenheimer on the Occasion of His 65th Birthday	Other

Data on citation counts were collected from WoS on 13.06.17

### Gender of articles’ subject

**Fig. 3:**
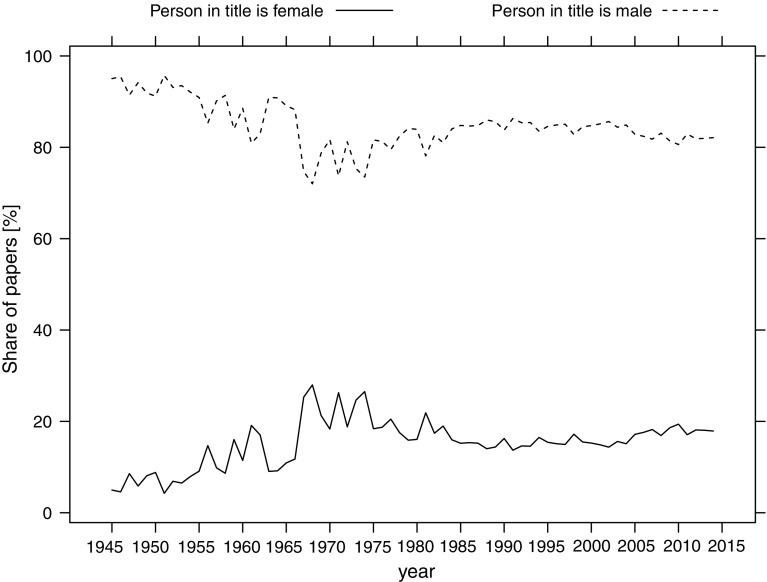
Share of women and men in the titles of biographical articles during 1945–2014. Only those articles are considered for which the algorithm managed to classify the gender of a person in the title

The classification algorithm helped us classify that out of 190,350 biographical articles,
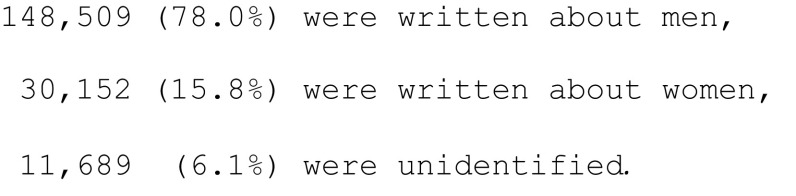


At the beginning of the studied period, most articles (over 90%) were about men. The share of articles about women had been slightly increasing till around the seventies of the twentieth century, when it stabilized at around 20% (Fig. 3). Such an increase in the share of articles about women was likely related to the movement toward gender equality in workforce in the 1960th and 1970th. However, the stable number of articles published about women from 1970th up to the present time suggests that little progress have been made in the appreciation of the contribution of women.

**Fig. 4:**
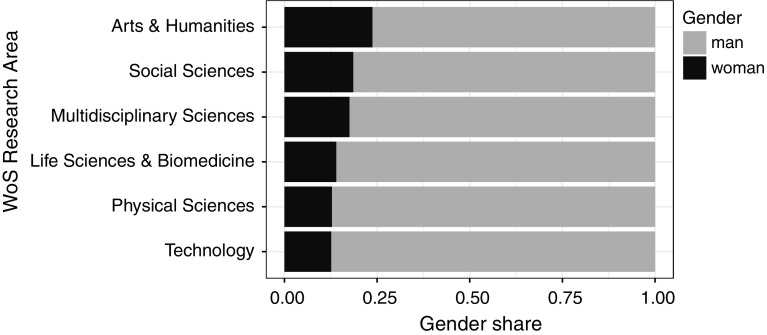
Share of articles about women and men in the WoS Research Areas

The highest share of articles about women was in Arts & Humanities (almost 24%), Social Sciences (over 18%), and Multidisciplinary Sciences (over 17%). The lowest share was in Life Sciences & Biomedicine (14%) and Technology and Physical Sciences (both over 12%) (Fig. 4).

**Fig. 5:**
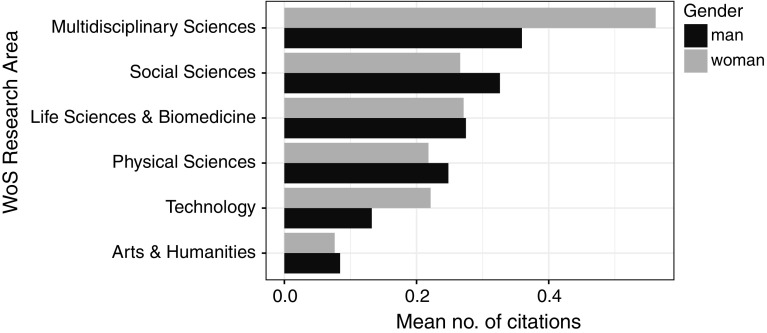
Mean number of citations per article about women and men for the WoS Research Areas

The articles about women were slightly less often cited (with the mean number of citations of 0.19) than those about men (0.21). Interestingly, the “unidentified” articles were more often cited (0.24). In Multidisciplinary Sciences and Technology, the articles about women were cited more often than those about men, even though in Technology the share of articles about women was the lowest. In Social Sciences and Physical Sciences, the situation was opposite. In Arts & Humanities and Life Sciences & Biomedicine, the mean number of citations per article was similar for the men and the women (Fig. 5).

#### What’s in there? Classification of biographical articles

A quarter of the sample of 750 biographical articles were articles in honor of an alive individual while 70% were in honor of a deceased person. We failed to collect sufficient information about nearly 5% of the articles, so we did not classify them. Unlike in the citation analysis, none of the 750 articles was wrongfully classified by WoS as a biographical article.

**Fig. 6:**
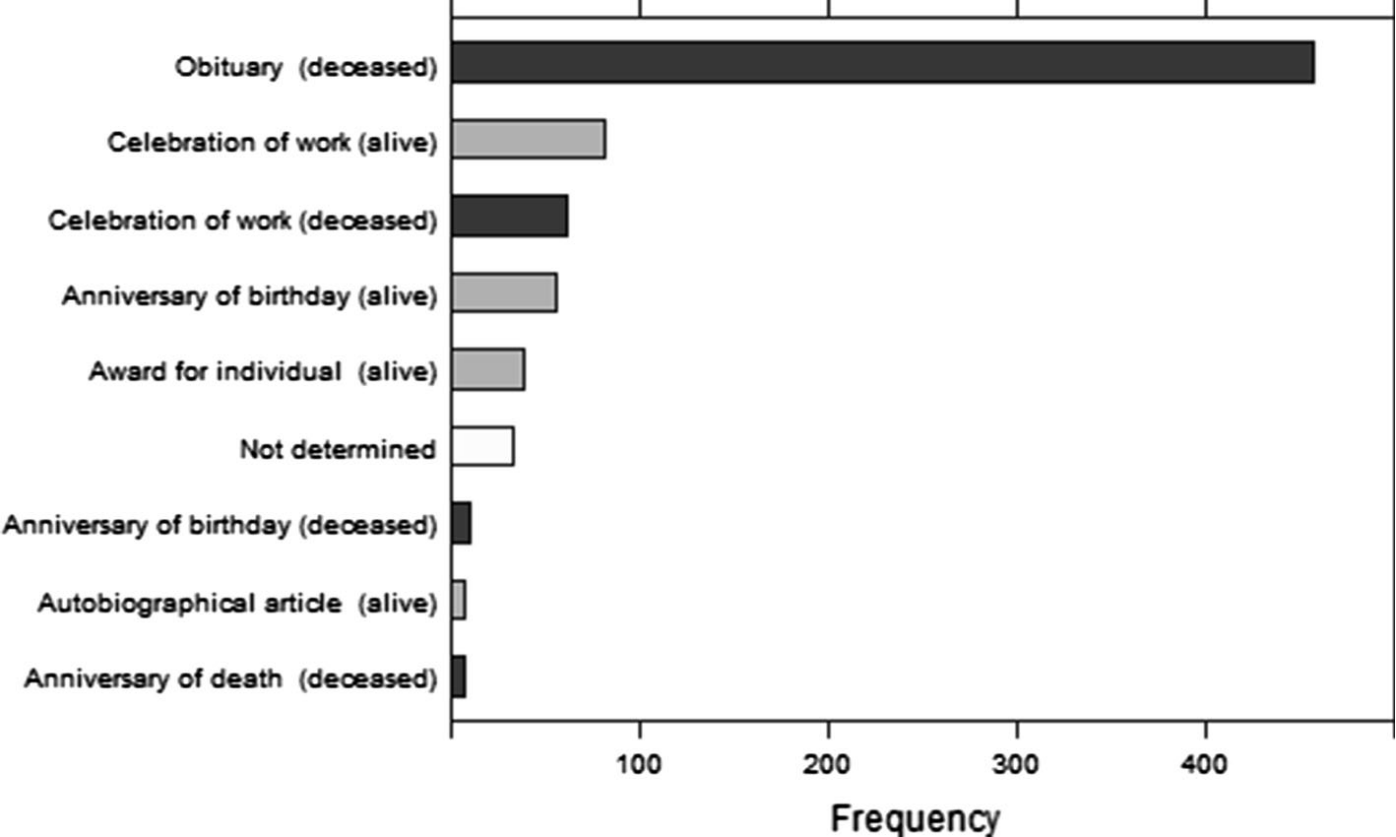
Frequency of analyzed subcategories

The sample was stratified, with 250 biographical articles from three strata being represented by the periods of up to 1984, 1985–1999, and from 2000 to 2014. The periods did not differ in the distribution of the subcategories (results not shown), which suggests biographical articles have not changed, as a document type, throughout this period, even if their number has been changing (Fig. 6).

Popularity of biographical articles categories differed across Web of Science research areas. Table 4 represents shares of different categories of articles in research areas.

**Table 4:** Shares of biographical articles across Web of Science research areas

Research area		Life Sciences & Biomedicine (%)	Arts & Humanities (%)	Physical Sciences (%)	Social Sciences (%)	Technology (%)	Multidisciplinary Sciences (%)	Total (%)
Category								
Obituaries (deceased)		45	26	11	11	7	1	100
Celebration of work (alive)		30	37	9	9	12	4	100
Celebration of work (deceased)		56	20	7	8	10	0	100
Anniversary of birthday (alive)		36	18	23	4	16	4	100
Award for individual (alive)		42	13	18	11	13	3	100
Anniversary of birthday (deceased)		70	10	20	0	0	0	100
Autobiographical article (alive)		43	0	57	0	0	0	100
Anniversary of death (deceased)		33	33	17	17	0	0	100

Obituaries represented the majority of biographical articles (458 out of 750, 61%). Over 45% of them were in Life Sciences & Biomedicine. Celebration of work was more common for alive people; most such articles were in Arts & Humanities (37%) and Life Sciences & Biomedicine (30%). Celebration of work was common for dancers, singers, and medical doctors in such journals as *Ballettanz, Dance Magazine, Popular Music, Opera News, Lancet, and British Medical Journal*. Celebration of work of deceased people was most common in Life Sciences & Biomedicine. Articles celebrating birthday anniversaries of alive people were most popular in Life Sciences & Biomedicine, followed by Physical Sciences, Arts & Humanities and Technology. Articles about awards for individuals were mainly from Life Sciences & Biomedicine (42%), followed by Physical Sciences (18%). All seven autobiographical articles in the sample were from Physical Sciences and Life Sciences & Biomedicine.

Seventy-seven percent of obituaries were about men and 15% about women; others were not classified (8%). A similar gender distribution was for anniversary of birthday (alive), award for individual, and celebration of work for both alive and deceased individuals. All anniversaries of birthday of deceased people as well as all anniversaries of death were only about men. Out of 7 autobiographical articles two were about woman.

### Authors

**Fig. 7:**
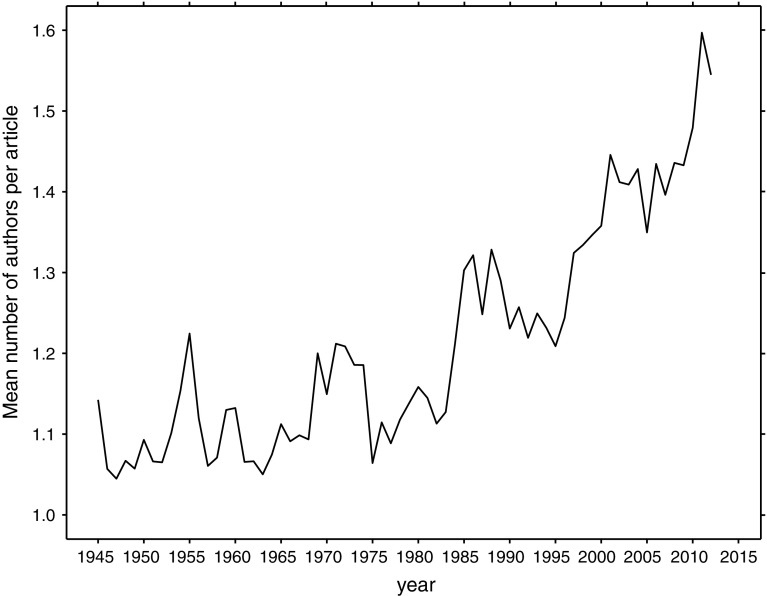
Mean number of authors of the biographical articles across the studied period

We have also analyzed the authors of the biographical articles. The 190,350 articles were written by 251,908 authors. The mean number of authors of biographical articles ranged between 1.04 and 1.6 and have been increasing since 1984 (Fig. 7). The highest number of authors (145) was for a tribute article to the Nobel Prize laureate Robert Geoffrey Edward. A supplement to the main issue of *Reproductive Biomedicine Online*, this article was atypical, being a collection of 130 tributes (Kamal et al. 2011). Fourteen of the top 20 articles with the highest number of authors were published in the Russian language and in Russian journals; the remaining six were written in English. Two Russian journals had seven and six articles with over 50 authors. Among them, fifteen articles mentioned men in their titles, and four mentioned women; the algorithm failed to identify one person’s sex; we checked it, and it was a man.

**Fig. 8:**
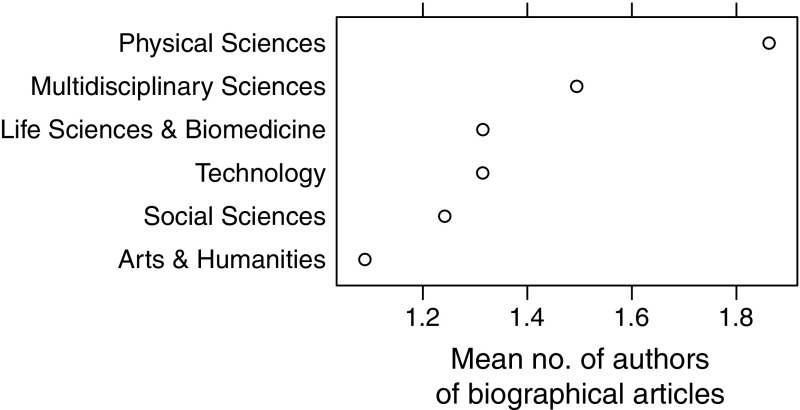
Mean number of authors of biographical articles for WoS research areas

The highest mean number of authors was for Physical Sciences while the smallest, for Arts & Humanities (Fig. 8). This result confirms what we know about Physical Sciences: that scientific articles in physics often have many co-authors (Iglič et al. 2017; Ioannidis, Klavans and Boyack 2016).

**Fig. 9:**
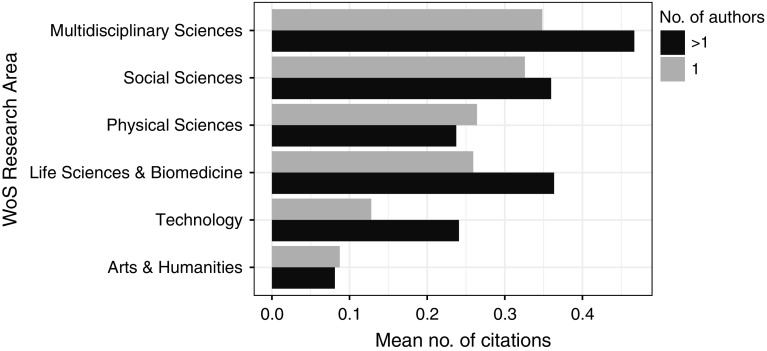
Mean number of citations per article for articles by one or more authors in WoS research areas for the studied period

In Multidisciplinary Sciences, Social Sciences, Life Sciences & Biomedicine, and Technology, biographical articles written by more than one author were more often cited than those written by one author (Fig. 9). In Physical Sciences and, to a smaller degree, in Arts & Humanities, the situation was opposite.

Based on the first names of the authors, we classified the authors’ gender for 60.1% of the authorships (see the glossary to recall what the term means). For 39.9% of the authorships, the classification was impossible for two reasons: (i) authors in over 11% authorships were anonymous, and (ii) initials instead of first names were given for many non-anonymous authors. Note when an author wrote *k* articles, we counted this author *k* times. Table 4 shows the authors’ gender (Table 5).

**Table 5:** Gender of authorships of biographical articles, classified based on first name

Author’s gender	Count	Share (%)
Woman	37,275	15
Man	114,083	45
Unknown	100,550	40
Total	251,908	100

**Fig. 10:**
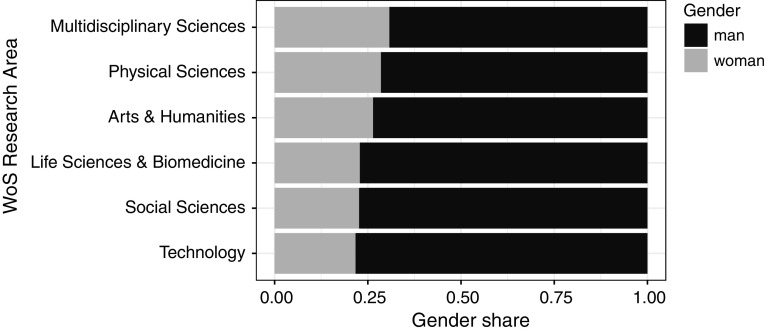
Share of male and female authors in WoS research areas

Among those authorships for whom we classified the gender, the women constituted 24% of authors while the men, 76%. The female authors had the highest share (around 30%) in Multidisciplinary Sciences and the lowest (around 20%) in Technology (Fig. 10).

### Authors and articles

**Fig. 11:**
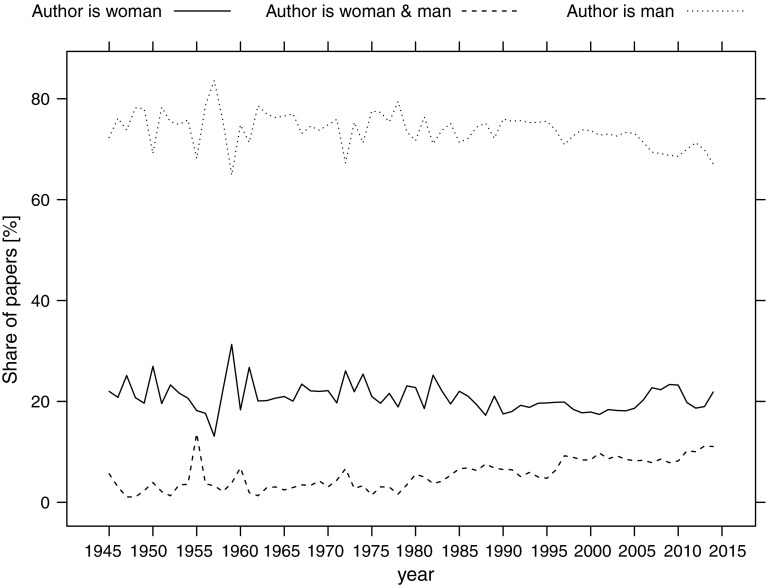
Share of biographical articles published by men, women and man-woman teams. Articles for which the authors were unidentified are not included

We have classified the biographical articles into those written by men, women, and both men and women (Table 6). For over 40% of articles, we were unable to identify the authors’ gender. Of the classified articles, 72% were written by men, 20% by women, and only 7.5% by a team of men and women. This classification was stable over the studied period (Fig. 11).

**Table 6:** Classification of articles by authors’ gender

Authors’ gender	No. of articles	Share of articles among the classified ones
Male	81,411	72.2%
Female	22,872	20.3%
Male and female	8468	7.5%
NA	77,599	–
Total	190,350	–

**Fig. 12:**
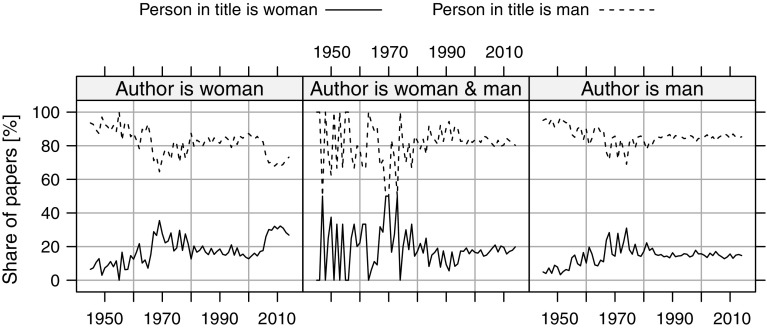
Share of articles written by women, men, and men-women teams about men or women, during 1945–2014. Included are only those articles for which we were able to identify gender of the authors and of the subject

Above, we have analyzed the biographical articles in two contexts: who wrote them and about whom they were written. Now, let us join the two contexts and analyze who wrote about whom (Fig. 12). As we already know, most articles were about men. This phenomenon did not depend on who wrote the articles (Fig. 12). After 2005, there was a peak in the articles about women written by women, but it lasted only for a few years. The trend for the male authors has been stable since the eighties, with the highest share of the articles written about men. A similar situation was for articles written by both women and men, though in a few earlier years, such teams published a similar number of biographical articles about men and women.

**Fig. 13:**
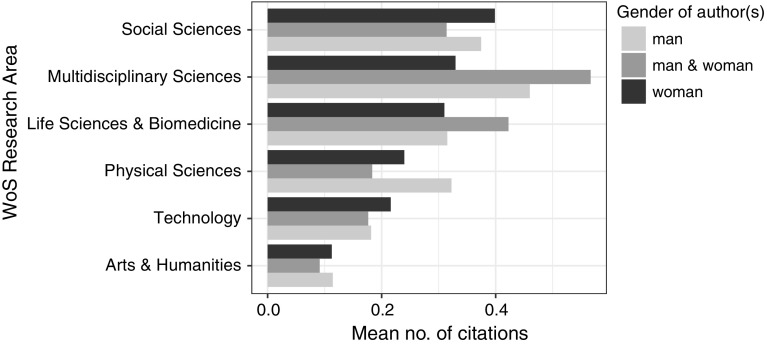
Mean number of citations per article authors by men, women and men-women teams, in the WoS research areas

The articles written by men were least often cited (with mean citation count of 0.25) while those published by men-women teams were most often cited (0.29); the articles published by women had, on average, 0.27 citations per article. The WoS Research Areas differed in citation patterns (Fig. 13). For Multidisciplinary Sciences, Physical Sciences, Life Sciences, and Arts & Humanities, the articles written by women were more often cited than those written by men. However, for Social Sciences and Technology, the articles written by men were most often cited. For Multidisciplinary and Life Sciences & Biomedicine, the articles written by men-women teams were most often cited.

## Conclusions and implications

Journals often use different names for article types than WoS does, hindering scientometrics research. Much more damaging, though, can be misclassification of articles into document types by WoS: such misclassification can affect the evaluation of journals and scholars (Harzing 2013). We found that biographical articles are seldom misclassified in WoS—although we did find such a misclassification among the most-cited articles, we did not among the 750 articles in the sample.

As mentioned in Introduction, previous studies on obituaries analyzed articles published in popular media; the majority of them presented content analysis of samples of obituaries. Till now, however, no one has attempted to analyze in detail biographical articles published in scientific journals—which is surprising, given the number of such articles. This gap led us to conduct the present research, which aimed to analyze biographical articles—including obituaries—published in scholarly journals indexed in Web of Science.

Most biographical articles do not directly contribute to the development of science. We do believe, however, that they do deserve attention—because they deal with one of the most valuable aspects of science development: the excellence of human mind. These over 190,000 biographical articles celebrating distinguished individuals constitute a rich source of information about the science world.

Thanks to analyzing biographical articles over the last 70 years, we were able to study various aspects of the development of this type of article. Some of such aspects were trends over time and across science disciplines related to article number, citation impact, variety in contents, and gender equality in article topics and authors.

Over time, the number of biographical articles in WoS has been increasing, including not only obituaries, but also job anniversaries, birthday celebrations, and commemorations of individuals. Most biographical articles were published in Life Sciences and Biomedicine, but the highest mean number of citations were in Social Sciences (although the between-area differences were rather small). This result is astonishing because regular scientific articles in Life Sciences and Biomedicine are much more frequently cited than those in Social Sciences. Dealing with people, not with scientific observations, however, biographical articles are governed by different rules than scientific articles are. Social scientists are likely more apt to write and read about people, and so they might be more apt to cite such articles. Among the top ten most often cited articles, however, only one was in Social Sciences; seven were from Life Sciences and Biomedicine, one from Technology, and one from Physical Sciences. We believe that some of these articles gained such high popularity (represented by many citations) because of their contents: they were more of review articles than biographies in the traditional meaning (e.g. Murdoch 1994; Westphal 1975).

The number of co-authored biographical articles (that is, written by more than one author)—in particular of those written by man-woman teams—has been increasing since the 1980s. This observation reinforces the leap towards collaboration in science (e.g. Adams et al. 2005; Persson et al. 2004; Glänzel 2002).

Over the studied period, the share of biographical articles commemorating men and women were stable, the overall representation of women in titles of biographical articles being 20%. The only exceptional period was several years in the 1970s, when this number increased by about 10 percentage points. Arts and Humanities had the highest share (24%) of articles about women; Life Science & Biomedicine, Physical Science, and Technology were at the opposite pole, with around 13% of biographical articles about women. From the analysis of the articles’ subjects and authors, a clear picture follows that the gender of an author is not related to the subject’s gender: both men and women wrote more about men than about women. Our research shows that more has to be done to commemorate women. *The New York Times*’ input is worth noting, with their weekly postings of obituaries of overlooked individuals in the past, mostly women and representatives of minority groups (The New York Times, 2018).

**Fig. 14:**
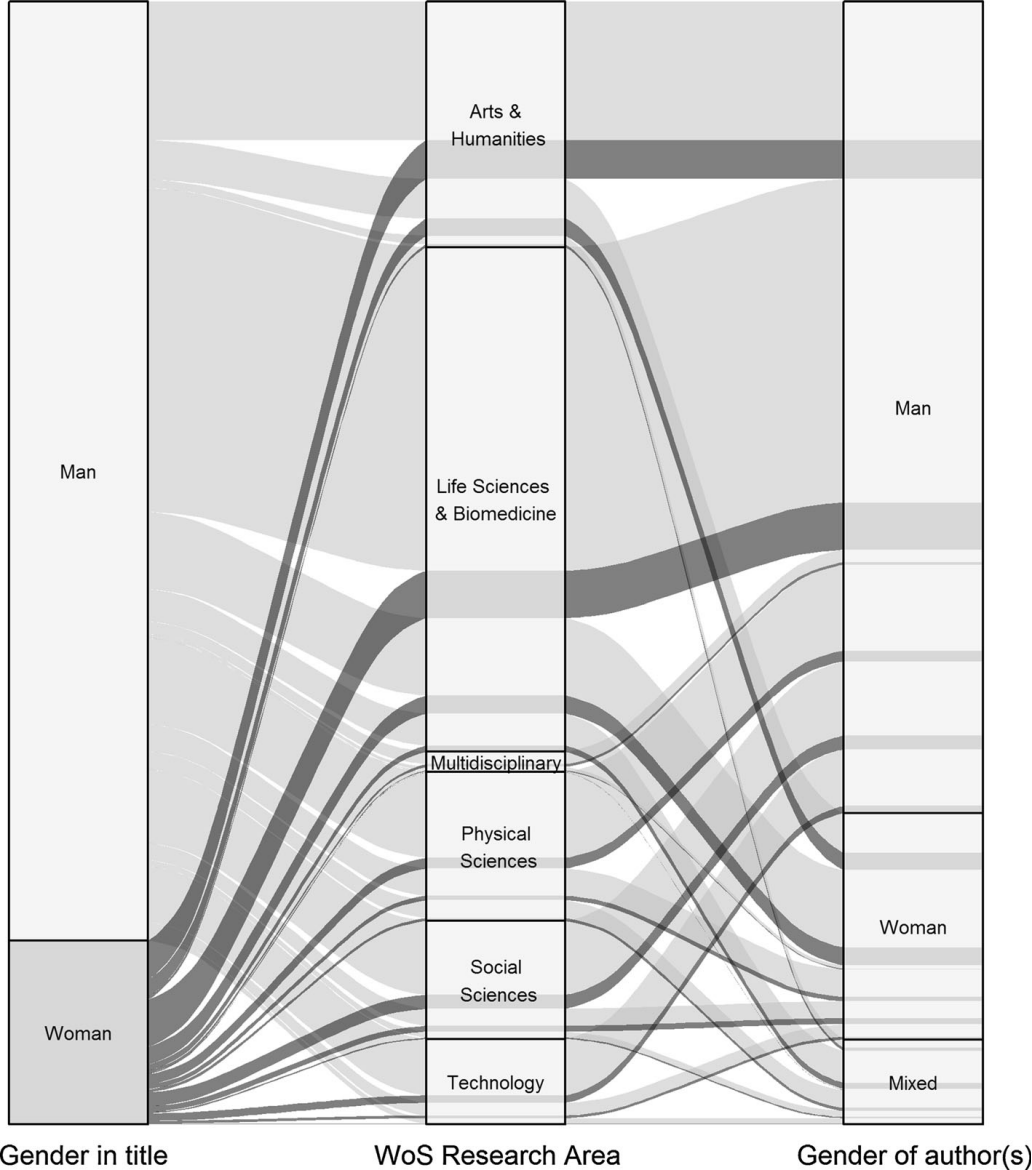
Alluvial plot showing who writes about women and men in different research areas

Figure 14 summarizes the relationships between the authors’ and the subjects’ genders in the WoS research areas. It clearly shows that, irrespective of the area, women are underrepresented in biographical articles in both roles: of their authors and of their subjects.

We based our analysis on an external name-gender reference dataset (the *genderize.io* database, Strømgren 2016). One limitation of using this database is that it was collected from social media networks. Declarative data that such networks collect from its users do not have to conform to reality. Thanks to the huge number of observations the *genderize.io* database consists of, however, the uncertainty related to the data’s declarativity should be negligible. Our validation tests of the database confirmed this thesis.

Web of Science assigns each article it indexes to one of many article types, two of which are “biographical items” and “items about and individual.” This assignment is not error-free, however, and mistakes happen (Harzing 2013). Our analysis showed that such misclassification of articles as biographical ones happen, but happen infrequently. It might be interesting to study possible reasons for such mistakes.

This paper opens new avenues for future research. What are the reasons behind the drop in the number of biographical articles over time? What is their importance to the scientific community? Do scientists read such articles? Why they do or don’t? We hope this paper will trigger research on the still understudied topic of scientometrics, that is, biographical articles.
